# Keeping Emotions in Mind: The Influence of Working Memory Capacity on Parent-Reported Symptoms of Emotional Lability in a Sample of Children With and Without ADHD

**DOI:** 10.3389/fpsyg.2018.01846

**Published:** 2018-10-02

**Authors:** Daniel André Jensen, Marie Farstad Høvik, Nadja Josefine Nyhammer Monsen, Thale Hegdahl Eggen, Heike Eichele, Steinunn Adolfsdottir, Kerstin Jessica Plessen, Lin Sørensen

**Affiliations:** ^1^The Department of Biological and Medical Psychology, University of Bergen, Bergen, Norway; ^2^K.G. Jebsen Centre for Research on Neuropsychiatric Disorders, Bergen, Norway; ^3^Betanien District Psychiatric Center (DPS), Bergen, Norway; ^4^Department of Clinical Medicine, University of Bergen, Bergen, Norway; ^5^Division of Psychiatry, Haukeland University Hospital, Bergen, Norway; ^6^Division Mental Health Services, Akershus University Hospital, Lørenskog, Norway; ^7^Child and Adolescent Mental Health Center, Copenhagen, Denmark; ^8^Division of Child and Adolescent Psychiatry, Department of Psychiatry, Lausanne University Hospital, Lausanne, Switzerland

**Keywords:** working memory, attention-deficit/hyperactivity disorder, emotional lability, emotion regulation, letter–number sequencing

## Abstract

Emotional lability (EL) often co-occurs with attention-deficit/hyperactivity disorder (ADHD) in children. However, difficulties of regulating intense emotions in ADHD are still poorly understood. We investigated the potential role of working memory (WM) as a protective factor against EL in children with ADHD by building on models describing the close relationship between WM and regulation of emotions. The parents of 41 children with ADHD and 34 typically developing children (TDC) filled out the emotional control scale (ECS) from the Behavior Rating Inventory of Executive Functioning and the child behavior checklist (CBCL). The children themselves completed the backward conditions of the digit span (DS) and spatial span (SS) tasks as well as the letter–umber sequencing (LNS) task. The results of a stepwise regression analysis confirmed the negative relationship between parent reported EL measured using the ECS and scores on the LNS, when controlling for symptoms of ADHD and oppositional defiant disorder (ODD). WM thus seems to be important for the ability of the children to express emotions in an adaptive and flexible way. We therefore suggest that a poorer WM capacity, which is often found in children with ADHD, may be a predictor of high levels of EL.

## Introduction

Attention-deficit/hyperactivity disorder (ADHD) is a frequent neurodevelopmental disorder present in around 5% of children (Polanczyk and Jensen, 2008; Willcutt, 2012). Problems of self-regulation associated with the disorder include difficulties in both cognitive (Willcutt et al., 2005) and emotional (Shaw et al., 2014) control functions (see Nigg, 2017). Typical difficulties include a reduced performance on working memory (WM) tasks, which measure the capacity to monitor and modulate incoming information (see the meta-analyses of Martinussen et al., 2005; Kasper et al., 2012). At the same time, parents of children with ADHD tend to report that their children have problems controlling their emotional expressions (Skirrow et al., 2009). This has been described as emotional lability (EL; e.g., Sobanski et al., 2010), which includes frequent expressions of high intensity (negative) emotions (Skirrow et al., 2009; Shaw et al., 2014). Such difficulties can be assessed with parent reports on the emotional control scale (ECS) of the Behavior Rating Inventory of Executive Function that measures the ability to modulate emotional responses, with high scores indicating a high level of EL or explosiveness (BRIEF; cf. page 18 of Gioia et al., 2000). The ability to monitor and modulate incoming information (WM capacity) is believed to be very important for the adaptive perception, experience, and expression of emotions (i.e., level of EL) (e.g., Gross, 2002; Sheppes and Gross, 2011; Sheppes et al., 2014; Smith and Lane, 2015).

Baddeley’s (Baddeley, 1986, 2000, 2002) WM model can be used to understand the role of WM in emotional experiences and the modulation of these experiences. He describes WM as a hierarchical system comprising a central executive that regulates and controls the storing (the phonological loop and the visuospatial sketchpad) and integration (the episodic buffer) of information from multiple modalities. The central executive is, as such, essential for monitoring and modulating incoming information by regulating the allocation of attention in accordance with goal-oriented behavior. A higher WM capacity can help a child to modulate an emotional reaction by taking into perspective the situational expectancies (e.g., such as downregulating the emotional impact of a situation; Knudsen, 2007). Previous studies have shown that the contribution of the capacity to modulate the meaning and importance of emotional experiences is important in pursuing goal-oriented behavior (Gross, 2002; Sheppes and Gross, 2011; Bridgett et al., 2013; Sheppes et al., 2014; Smith and Lane, 2015). Gross (2002), Sheppes and Gross (2011), McRae et al. (2012), and Sheppes et al. (2014) have focused on the role of verbal WM in the experience, expression and regulation of emotions in typically developing adults and concluded that the ability to cognitively reappraise experiences eliciting negative emotions is related to better performance on verbal WM tasks. Typically, participants have been exposed to emotional stimuli with varying valence, such as emotion eliciting images, with the instruction to actively reduce the emotional impact of the stimuli by constructing alternative interpretations. One study finding supporting evidence in typically developing, young adults showed that the distribution of pre-made reappraisals, assumed to decrease the cognitive cost of reappraisal, increased the ability to down-regulate the intensity of negative emotions and thus facilitated the reappraisal process (Sheppes et al., 2014). Similarly, research investigating the role of cognitive control in emotional experience from a developmental perspective (i.e., based on the model of Posner and Rothbart, 2000), have also implicated the importance of verbal WM capacity (Bridgett et al., 2013). This is in line with work suggesting a relationship between reduced WM capacity, as part of executive functioning, and emotional difficulties in children with ADHD (e.g., Nigg et al., 2004; Sheppes et al., 2015).

The high prevalence of EL difficulties in children and adults with ADHD (Skirrow et al., 2009) has been noted over time (Wender, 1972), and has been included as an associated feature to ADHD in the fifth edition of the Diagnostic and Statistical Manual of Mental Disorders (DSM-5; American Psychiatric Association, 2013). Previously, efforts to explain this association have predominantly focused on poorer inhibitory control (Barkley, 1997) and high levels of oppositional defiant disorder (ODD; e.g., Sobanski et al., 2010) as predictors of EL.

Only one prior study (Banaschewski et al., 2012) has, to the best of our knowledge, investigated how inhibitory control and WM relate to EL in ADHD. They found no significant association between these functions and parent-reported levels of EL after controlling for ADHD symptoms. However, the WM task applied, the digit span (DS), is probably not as sensitive as other measures of verbal WM in assessing the capacity to modulate incoming information (i.e., simple reversal of a single stimulus category may not be sufficiently cognitively demanding; Shelton et al., 2009; Kasper et al., 2012). We therefore wanted to investigate a possible link between verbal WM and parent-reported EL by including a WM task that is assumed to place a higher load on the modulation of incoming information than the digit span, namely the letter–number sequencing (LNS) task (e.g., a “complex” task; Shelton et al., 2009). The LNS requires the participant both to remember (store, i.e., the phonological loop) and to sequence the digits and letters that are presented according to numerical and alphabetical order (integrating stored information and modulating it according to knowledge of the alphabet, i.e., the episodic buffer). Thus, introducing a greater processing demand and reliance of the central executive than simple reversal. It is important to note that WM, together with inhibitory control and cognitive flexibility, are suggested to comprise the subfunctions of cognitive control (Miyake et al., 2000). WM is thus shown to load on inhibitory control, however, not on cognitive flexibility (see Miyake and Friedman, 2012). Following Baddeley (1986) model, the central executive acts as an inhibitory control component. However, there is ample evidence of the importance of WM – and not inhibitory control alone – in several emotion regulation strategies (Smith and Lane, 2015), including cognitive reappraisal (McRae et al., 2012). This may be because these processes involve multiple components of WM. In addition to inhibitory control (i.e., central executive), the information is modulated by holding it in temporary storage (i.e., the phonological loop and the visuospatial sketchpad) at the same time as the information is integrated with existing knowledge and experience (i.e., the episodic buffer) (Baddeley, 2000, 2002).

Therefore, based on the theories of Gross (Gross, 2002; Sheppes and Gross, 2011; Sheppes et al., 2014) and Posner and Rothbart (Posner and Rothbart, 2000; Rueda et al., 2005; Rothbart et al., 2011; Bridgett et al., 2013) as well as a recent review (Smith and Lane, 2015), we expected an inverse relationship between verbal WM and parent reported difficulties related to EL. To examine this hypothesis we used three WM tasks – the DS and SS which can be described as “simple reversal” verbal and visuospatial span tasks, respectively, and the LNS which can be described as a complex verbal WM task – and only expected verbal WM capacity to associate with EL, and then only with the WM task with the highest load on modulation of incoming information (i.e., the LNS; Shelton et al., 2009). We also wanted to explore whether this association was independent of parent reported symptoms of ADHD and ODD, and diagnostic status. As the reviewed studies (e.g., Gross, 2002; Sheppes and Gross, 2011; McRae et al., 2012; Sheppes et al., 2014) indicate an inverse relationship between EL and WM capacity in healthy samples, this inverse relationship may not distinguish between the ADHD group and the typically developing children (TDC). However, we expected higher levels of parent reported EL symptoms and a poorer WM capacity in the ADHD group than among the TDC. Furthermore, due to the noted association between WM and inhibition, as well as prior theories emphasizing the importance of difficulties related to inhibition, we also conducted supplementary analyses to investigate whether inhibition would be a significant contributor to the current results (see **Supplementary Materials**).

## Materials and Methods

### Participants

The current study included 75 children between 8 and 12 years old, and consisted of 41 children with an ADHD diagnosis and a control group of 34 TDC. There were no group differences in sex or age distributions between the two groups (**Table 1**).All participating children had a full-scale intelligence quotient (FSIQ) above 75, however, the children with an ADHD diagnosis had lower FSIQ than the TDC. The study was carried out with the approval of the Regional Ethical Committee for Western Norway (REK-Vest), and written informed consent in accordance with the Declaration of Helsinki was obtained from all parents.

**Table 1:** Descriptive characteristics of the sample.

	TDC		ADHD		Between-group effects			
	*M*	SD	*M*	SD	*F/chi square*	*Df*	*p*	*Post hoc*
Age (years)	9.47	1.08	9.65	1.25	0.43	1/72	ns	
FSIQ	105.76	11.07	91.15	7.33	4.71	1/72	<0.001	TDC > ADHD
GAI	111.94	12.47	95.60	8.85	2.85	1/72	<0.001	TDC > ADHD
ADHD	1.00	1.33	9.34	2.47	310.16	1/72	<0.001	TDC < ADHD
ODD	0.76	1.28	4.49	2.95	46.88	1/72	<0.001	TDC < ADHD
ECS	12.68	3.21	19.75	5.63	41.99	1/72	<0.001	TDC < ADHD
DS	6.74	1.69	6.03	1.33	4.08	1/72	<0.05	TDC > ADHD
SS	7.41	2.00	5.80	1.42	16.32	1/72	=0.001	TDC > ADHD
LNS	15.85	4.05	12.55	3.62	13.72	1/72	=0.001	TDC > ADHD
Boys/Girls	20/14		29/11		1.54	1	ns	
ODD-diagnosis (number/total)	0/34		16/40		17.35	1	<0.001	Pearson *X^2^*

*FSIQ, full scale IQ; GAI, general ability index; ADHD, scores on the attention deficit/hyperactivity problems scale of the CBCL; ODD, scores on the oppositional defiant problems scale of the CBCL; DS, score on the digit span backward condition; SS, score on the spatial span backward condition; LNS; score on the letter–number sequencing task; TDC, typically developing children; ODD-diagnosis, oppositional defiant disorder-diagnosis.*

Children with a suspected ADHD diagnosis were referred from outpatient child and adolescent psychiatric clinics serving the municipality of Bergen, Norway. A control group of TDC was recruited from schools in geographical areas overlapping with the areas served by the above mentioned outpatient clinics.

Exclusion criteria for both groups were an existing ADHD diagnosis and prior use of psychostimulant medicine due to the wish to study cognitive functions that had not been modulated by treatment effects (Eichele et al., 2016; Plessen et al., 2016; Sørensen et al., 2017). Further exclusion criteria were, suspicion of an autism spectrum disorder, or a prior head injury with loss of consciousness. The diagnosis of ADHD was given following the algorithm of the “Schedule for Affective Disorders and Schizophrenia for School-Age Children – Present and Lifetime Version” (K-SADS-PL; Kaufman et al., 1997). Clinical professionals interviewed the children and their parents using the K-SADS-PL, and a board consisting of a child psychiatrist and a clinical psychologist finally confirmed the diagnostic evaluations. Only children with a primary diagnosis of ADHD were included in the clinical group (n = 41), 26 children fulfilled the diagnostic criteria for the combined subtype, 12 had the predominantly inattentive subtype, and three the hyperactive/impulsive subtype on the basis of a best estimate diagnosis reviewing all available materials (Leckman et al., 1982). Comorbidities affected several of the participating children. Among the children with ADHD, ODD was the most common comorbidity (n = 17) with three of these children also fulfilling the criteria for a conduct disorder. Furthermore, 15 of the children with ADHD also fulfilled the criteria for an anxiety disorder and three the criteria for a tic disorder. One of the TDC fulfilled the criteria for a specific phobia. FSIQ was assessed using the Wechsler Intelligence Scale for Children – Fourth Edition (WISC-IV; Wechsler, 2003). The general ability index (GAI) score was also included as a measure of intellectual level in the current study, because WM scores are included in the calculation of the FSIQ scores (**Table 1**).

### Working Memory

Working memory was assessed with the backward conditions of the DS and the spatial span (SS) tasks, as well as the LNS task (Kaplan et al., 2004; Wechsler, 2003). In the backward conditions of the DS and the SS, children are instructed to recall and reproduce a list, or touch blocks, in the opposite order of that presented by the examiner (i.e., for the DS the examiner may read the sequence 2-7-1 and the child is to respond by reversing this sequence into 1-7-2), whereas the LNS requires the children to recall, rearrange, and reproduce a sequence of letters and numbers presented aloud by the examiner by first repeating the numbers in ascending order and then the letters in alphabetical order (i.e., the sequence E-1-F is to be rearranged into 1-E-F; Kaplan et al., 2004). The DS and SS have been described as “simple” span tasks (i.e., even though the tasks include reversal of stimuli this may not be sufficiently demanding to categorize such tasks as encompassing a high load on the central executive component of WM), whereas the LNS is the clinical measure which is most closely associated with laboratory measures of WM (i.e., additional processing of the stored information is required to correctly sort numbers by size and letters by alphabet placement; Shelton et al., 2009; Kasper et al., 2012).

### Emotional Lability (EL)

Emotional lability was measured with parent information on the emotional control subscale from the Behavior Rating Inventory of Executive Functioning, which “addresses the manifestation of executive functions within the emotional realm and measures a child’s ability to modulate emotional responses. Poor emotional control can be expressed as EL or emotional explosiveness” (Gioia et al., 2000, p. 18). This subscale asks the parents how they experience their children typically acting when they are upset, angry, or sad. Each item is evaluated according to a Likert-scale with three response alternatives: “often” (score 3), “sometimes” (score 2), or “never” (score 1). Internal reliability, as estimated by Cronbach’s alpha, is high (0.92; Gioia et al., 2000), also in a Norwegian sample in a comparable age group (Ranging from 0.80–0.98 for all subscales; Sørensen et al., 2011), and in the current sample (0.94 for the ECS). In the linear statistical analyses, we used the raw scores to secure a higher variability in scores (i.e., standardized scores are centralized around the mean).

### Dimensional Symptom Scales of ODD and ADHD

We used the parent form of the child behavior checklist (CBCL), part of the Achenbach System of Empirically Based Assessment (ASEBA; Achenbach and Rescorla, 2001), to investigate the predictive validity of symptoms of ADHD and ODD on EL. The subscales of interest in the current study were the oppositional defiant problems scale (ODD symptom scale) and the attention-deficit/hyperactivity problems scale (ADHD symptom scale). The CBCL is a highly validated and reliable measures in this age group (Achenbach and Rescorla, 2001), and also for use with clinical populations, including children and youth with ADHD and comorbidities (Achenbach and Rescorla, 2001; Krol et al., 2006; Biederman et al., 2008).

### Statistical Analyses

All statistical analyses were conducted using IBM SPSS, version 25. Bivariate correlation analyses were conducted among all variables of interest. To test our main hypothesis, we conducted a linear stepwise regression analysis that included EL scores from the ECS as the dependent variable, and age, gender, symptoms of ODD and ADHD, GAI scores, and WM scores of DS, SS, and LNS scores as the independent variables. FSIQ was not included as it has been argued that controlling for it as a covariate is likely to distort findings (Dennis et al., 2009).

The stepwise regression analysis was followed by a moderation analysis as described by Kraemer et al. (2002) and Hayes (2012) building on the model of Baron and Kenny (1986). In our study this comprised a regression approach including the independent variable of the LNS scores (IV), a moderator variable of diagnostic status of ADHD versus TDC (M), and an interaction variable of the LNS scores by moderator variable of diagnostic status (IV × M) with the EL scores from the ECS as the dependent variable.

To investigate the potential influence of inhibition on the relationship between WM-scores and EL scores from the ECS we repeated the primary stepwise regression with the Stop-Signal Task score as an independent variable together with WM scores, symptoms of ADHD and ODD, age, and gender (see **Supplementary Materials**).

Missing data for one child each on ADHD symptoms, ODD symptoms, and GAI were replaced with the series mean. Furthermore, an inspection of the studentized residuals showed that one participant belonging to the group of children with ADHD was an outlier (Aguinis et al., 2013). This child’s data were therefore omitted from the analyses.

## Results

### Preliminary Results

Preliminary correlational analyses of the relationship between age and the variables of EL and the WM scores (LNS, SS, and DS), showed that age correlated significantly with the WM scores of SS and LNS (**Table 2**). Age did, however, not correlate with the DS scores. Gender appeared to only be significantly correlated with the DS scores and not with the other WM scores (LNS and SS). The parent-reported symptoms of EL, ODD and ADHD were not significantly correlated with either age or gender (See **Table 2**). All three WM scores of LNS, SS, and DS correlated significantly with each other.

**Table 2:** Correlations among the examined variables.

	1	2	3	4	5	6	7	8
(1) EL	–	−0.37^∗∗^	−0.31^∗∗^	−0.21^∗^	0.84^∗∗^	0.70^∗∗^	0.05	0.05
(2) LNS		–	0.53^∗∗^	0.49^∗∗^	−0.24^∗^	−0.40^∗∗^	0.33^∗∗^	0.03
(3) SS			–	0.36^∗∗^	−0.24^∗∗^	−0.47^∗∗^	0.36^∗∗^	−0.05
(4) DS				–	−0.09	−0.19	0.14	−0.21^∗^
(5) ODD					–	0.72^∗∗^	0.15	0.08
(6) ADHD						–	0.09	0.10
(7) Age							–	0.18
(8) Gender								–

*EL, score on the emotional control scale of the BRIEF; ADHD, score on the attention deficit/hyperactivity problems scale of the CBCL; ODD, score on the oppositional defiant problems scale of the CBCL; DS, score on the digit span backward condition; SS, score on the spatial span backward condition; LNS, score on the letter–number sequencing task. ^∗∗^Correlation is significant at the 0.01 level (1-tailed); ^∗^correlation is significant at the 0.05 level (1-tailed).*

### The Relationship Between WM Capacity and Parent-Reported Emotional Lability

All three WM scores of LNS, SS, and DS were inversely correlated with the parent-reported EL scores on the ECS. The forward linear stepwise regression model including the EL scores from the ECS as the dependent variable and the independent variables of age, gender, ADHD symptoms, GAI scores, and the WM scores of LNS, DS, and SS, showed that only symptoms of ODD and the LNS scores significantly predicted the parent-reported scores of EL on the ECS, and not age, gender, GAI, symptoms of ADHD, scores on the DS or scores on the SS (see **Table 3**).

**Table 3:** Results from the forward stepwise regression model showing the prediction of EL based on symptoms of ODD and LNS-scores.

		Model summary				ECS			
		Adjusted *R*^2^	ΔR	df	*p*	*B*	SE B	β	*p*
Model 1	ODD	0.70	0.71	1/72	<0.001	1.65	0.13	0.84	<0.001
									
Model 2	ODD	0.73	0.03	1/71	<0.01	1.56	0.12	0.80	<0.001
	LNS					−0.26	0.09	−0.18	<0.01

*ECS, score on the emotional control scale of the BRIEF; ODD, score on the oppositional defiant problems scale of the CBCL; LNS, Score on the letter–number sequencing task.*

**FIGURE 1:**
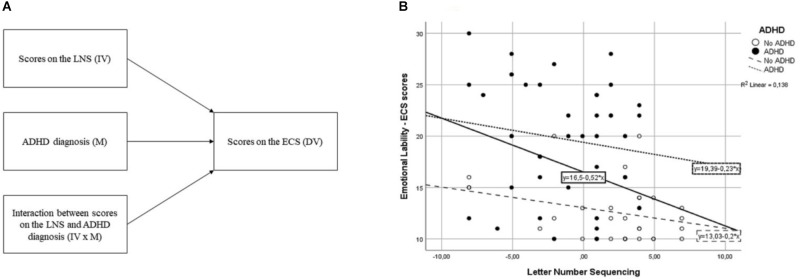
A graphical representation of the moderation analysis **(A)**, and a scatterplot showing the distribution of scores on the Letter–Number Sequencing (LNS) and Emotional Control Scale (ECS) as well as the equations describing the relationships between these for the group with attention-deficit/hyperactivity disorder (ADHD), the No ADHD as well as the whole sample **(B)**. Note: IV, independent variable; M, moderator; DV, dependent variable. Letter Number Sequencing = Centered scores on the Letter Number Sequencing task (i.e., individual scores minus the sample mean).

The results of the moderation analysis, investigating the interaction between ADHD diagnostic status and the LNS scores on the EL scores from the ECS, showed that there were no significant interaction between the LNS scores and diagnostic status (see **Figure 1**).

## Discussion

In line with our hypotheses, we found an inverse relationship between children’s verbal WM scores and parent reported EL. As expected, higher LNS scores were related to lower EL symptoms after controlling for parent-reported ADHD and ODD symptoms. The follow-up moderation analysis supported that this relationship was independent of diagnostic status, although the levels of both WM capacity and EL differed between the groups (i.e., children with ADHD had lower WM scores and higher EL scores than TDC, but the relationships between these scores did not significantly differ between groups).

The current findings are in line with previous studies in healthy samples (e.g., McRae et al., 2012; Sheppes et al., 2014) in that a lower WM capacity seems to be related to an increased probability of experiencing and expressing emotions in a way which is described as problematic by the children’s parents. Overall the current findings are therefore also in line with the model proposed by Gross and colleagues (Gross, 2002; Sheppes and Gross, 2011) and the previous findings that verbal WM is involved in expressing one’s emotions in an adaptive and goal-oriented way (Smith and Lane, 2015). The current findings can also be linked to similar findings from temperamental research showing an overlap between WM and efficient control of one’s emotions (Bridgett et al., 2013). This may suggest that a higher WM capacity acts as a protective factor against developing clinically significant difficulties in expressing one’s emotions (i.e., difficulties in controlling strong emotional outbursts – EL).

The findings may also be seen in connection with research on ADHD. There is a known relationship between ADHD and lower WM capacity (Martinussen et al., 2005). This lower WM capacity may be one of the factors contributing to the high prevalence of clinically significant levels of EL in this group (Skirrow et al., 2009; Sobanski et al., 2010). This is also in line with the suggestion that there may be a connection between difficulties in cognitive and emotional control (Nigg et al., 2004), although the current results cannot give any indication on the causal relationship between these difficulties. Important to note, though, is that the inverse relationship found between a lower WM capacity and higher levels of parent-reported ***EL*** did not appear to be restricted to children with ADHD. Rather this was shown to be a dimensional relationship true for the whole sample. The results, therefore, indicate that the findings regarding a relationship between WM and emotion regulation from studies on typically developing adult populations reviewed in this article are also applicable to children, both with and without ADHD. However, the children with ADHD showed poorer WM capacity on the LNS (and the SS) and a higher frequency of parent-reported EL symptoms than the TDC, indicating that the inverse relationship between EL symptoms and WM capacity may be more significant for their everyday functioning than for the group of TDC. Future studies may therefore want to investigate whether this holds true in other populations with elevated levels of EL, such as in children with anxiety disorders (Maire et al., 2017) and in adults with bipolar disorders (Phillips et al., 2003), borderline personality disorder (Schoenleber et al., 2016) and post-traumatic stress disorder (Schoenleber et al., 2018). Interestingly, in the current study, neither age nor gender affected the relationship between WM capacity as measured with the LNS and the level of parent-reported EL. This indicates that a poorer WM capacity seems to relate to higher levels of EL in general, independent of diagnosis, age and gender of the child. However, both an ADHD diagnosis and age showed an expected association with the performance on the WM tasks, with the exception that age did not correlate with the performance on the DS task. A differential effect of gender on the WM task scores also appeared, with boys scoring lower on the DS task compared to the girls, whereas such a difference did not appear on the SS and LNS. Previously, small age related improvements have been reported on the DS task in samples with similar age ranges as included in the current study (see Brocki and Bohlin, 2004; Lensing and Elsner, 2018). These findings seem to indicate two distinct periods of marked development in the ability to answer the task requirements of the DS, with one period ending around the age of ***8–9*** years, and the second commencing around 12***–***13 years of age. The period between these, spanning the age range of our participants, seems to be characterized by small developmental changes, and this may therefore be the explanation for the lack of association found between age and DS scores. With regard to gender effects on WM capacity in ADHD, previous studies show mixed results depending on the percentage of females included (Kasper et al., 2012). In studies with a more balanced gender distribution, as in the current study, smaller between-group effect sizes appear related to the WM capacity.

It is also worth mentioning some contrasts and similarities between the current findings and the findings of Banaschewski et al. (2012), as they found no association between WM and EL in children with ADHD. As stated in the introduction, we believe this may be due to the measure applied. The DS was the only measure of WM included in the study of Banaschewski et al. (2012), and our results support their conclusion that this measure is not closely associated with EL. However, we believe that the current results support the assumption that WM is in fact meaningfully associated with EL, and that WM as measured using the LNS specifically, seems to be particularly important. Another distinction between the two studies is the inclusion of a measure of ODD in the current work. Given that findings show that EL in ADHD seems to be more closely associated to ODD than to ADHD (Sobanski et al., 2010), we believe that the significance of the current findings even when controlling for symptoms of ODD further supports the notion that WM capacity may be an important protective factor against the development of EL. It is, however, worth noting the possibility that the close association between the LNS and EL may not be due to it being a specific measure of verbal WM, but of general WM capacity due to its’ higher demands on the modulation of information (Kasper et al., 2012).

The current findings highlight some interesting possible directions for future work. If the current results can be replicated in a larger sample we believe that this would also merit an investigation of whether measures of verbal WM could also be used to direct the implementation of clinical interventions aimed at reducing the impact of EL as an associated feature of ADHD, and at reducing the risk for comorbid difficulties related to EL (i.e., ODD; Sobanski et al., 2010). One potential intervention in this regard could be emotional WM training, which consists of a dual n-back task presenting a combination of auditory and visual stimuli where a majority of the stimuli have a negative emotional valence (Schweizer et al., 2011). Such training has been shown to have an effect on a frontoparietal network assumed to underlie both WM and affective control (Schweizer et al., 2013). Furthermore, results show that the effects of such training generalizes to traditional measures of emotion regulation (Schweizer et al., 2013). Another potential area of investigation is whether a screening of verbal WM can help inform the pharmacological treatment of ADHD. Building on the study by Cubillo et al. (2013) showing a differential effect of methylphenidate and atomoxetine, in combination with findings indicating an anatomical overlap between WM and self-regulation (e.g., Bridgett et al., 2015), it seems plausible to hypothesize that atomoxetine might be particularly beneficial for the subgroup of children with ADHD who also have a low WM capacity. This is due to differential effects showing that atomoxetine has a pronounced activating effect on the dorsolateral prefrontal cortex, a region which has been shown to be involved in both WM and executive attention (Bridgett et al., 2015).

### Strengths and Limitations

The current study had several important strengths and limitations. It employed neuropsychological measures which are often used in clinical practice (i.e., subtests from the WISC-IV and WISC-IV-Integrated) in combination with well validated and widely accessible questionnaires, thereby obtaining results which are available in, and transferable to, day-to-day clinical practice and may be replicated in many clinical settings. We also regard the use of dimensional analyses as a strength, as these allowed us to investigate the hypothesized pattern of results in both the children with ADHD and the TDC. This is in line with our expectations as the hypothesis was, to a large degree, based on studies of typically developing individuals.

The main limitations of this study are the limited sample size and the cross-sectional nature of our data. Due to these limitations all of our participants with high levels of EL belonged to the diagnostic group, thus limiting the generalizability of our conclusions. Furthermore, the use of cross-sectional data does not allow for investigation of the developmental ordering of the children’s difficulties, which would be highly relevant with regards to the model proposed by Nigg et al. (2004). A closer examination of whether the results reported here are mainly due to the use of a measure of verbal WM or a WM task with high demands on the modulation of information is also necessary to improve our understanding of the relationship between WM and EL. At the current time it could equally well be argued that a complex visuospatial WM task would be equally as predictive of parent reported EL scores, and a direct comparison of two complex WM tasks where one is assumed to be reliant on the verbal and one on the visuospatial component of WM would, therefore, help to clarify this issue. The results would also have been strengthened if the investigation had included a measure of task switching, as this executive function may associate with level of EL (e.g., Dickstein et al., 2007). There is also the issue of a significant difference in FSIQ between the two groups. Although this is common in studies of ADHD, and related to the known difference in WM capacity as well as likely to be related to test-taking behavior (Dennis et al., 2009), the findings should ideally be investigated in a sample with matched FSIQ scores. Lastly, the use of the same informant report when collecting information about symptoms of ADHD and ODD as well as EL may have reduced the statistical power of WM in the analyses (i.e., due to common-method variance; Richardson et al., 2009). Ideally, the investigation should be replicated with the inclusion of observer measures of emotional reactions to reduce the impact of this limitation.

## Conclusion

The current study found support for the hypothesis that WM is a protective factor against elevated levels of EL in children, thus supporting previous findings showing the importance of high (verbal) WM capacity in the adaptive display of emotions. The results, if replicated, may represent an approach to understanding the functional heterogeneity associated with ADHD.

## Author Contributions

KP and LS conceived and designed the study. MH, NM, HE, SA, KP, and LS acquired the data. DJ and LS analyzed and interpreted the data. DJ, MH, TE, KP, and LS wrote the manuscript. All authors contributed to manuscript revision, read and approved the submitted version.

## Conflict of Interest Statement

The authors declare that the research was conducted in the absence of any commercial or financial relationships that could be construed as a potential conflict of interest.
