# Supplementary material for: Keeping Emotions in Mind: The Influence of Working Memory Capacity on Parent-Reported Symptoms of Emotional Lability in a Sample of Children With and Without ADHD
Source: Front Psychol. 2018 Oct 2;9:1846. doi: 10.3389/fpsyg.2018.01846 (PMC6176092; doi:10.3389/fpsyg.2018.01846)
Supplement: Supplementary file 1 [file Table_1.pdf]

Supplementary material

As stated in the main text of this article previous theories and studies of EL in children with ADHD have focused on the potential role of inhibition (Banaschewski et al., 2012; Barkley, 1997). Furthermore, findings show a close relationship between WM and inhibition (Miyake & Friedman, 2012; Miyake et al., 2000). We therefore wanted to investigate whether the inclusion of a classical inhibition task would alter the reported relationship between LNS scores and EL.

Methods

Stop-Signal Task: The participating children performed the stop-signal task from the CANTAB (www.camcog.com; Chamberlain et al., 2011). This is a computer-based task consisting of two trial conditions. On go trials, the children were asked to indicate the direction of an arrow presented on the screen by pushing a corresponding button (i.e. right-hand button indicating the arrow pointed to the right and left-hand button indicating that it pointed to the left). In 25% of the trials, an auditory stop signal was presented (i.e. the stop signal), indicating that the children were to inhibit their responses. The measure of inhibitory control used in the current study was the stop signal reaction time (SSRT). This measure consists of the time interval (in milliseconds) between mean reaction times on go trials and the stop-signal delay (i.e. the timing of presentation of the stop signal). This interval is dynamically adjusted to ensure that all the children achieve a minimum of 50% correct responses on stop trials.

Statistical analyses:

We conducted follow-up analyses to investigate the potential role of inhibition. These included a correlational analysis and a forward linear stepwise regression analysis. The stepwise regression analysis included the parent-reported EL scores from the ECS as the dependent variable, and age, gender, symptoms of ODD and ADHD, stop-signal reaction time, DS, SS, and LNS scores as the independent variables.

Four additional children were excluded due to missing scores on the stop-signal task, three children with ADHD and one typically developing child.

Results:

The correlational analysis did not indicate any significant correlations between the stop-signal task and any of the other variables of interest (Supplementary table S1). Furthermore the results of the stepwise regression analysis did not change. Symptoms of ODD and LNS scores remained the only significant predictors of parent-reported scores on the ECS, whereas age, gender, scores on the DS and SS, symptoms of ADHD and SSRT were not found to be significant predictors (Supplementary table S2).

Discussion:

We interpret these findings to support the notion that WM is associated with parent reported symptoms of EL as measured by scores on the ECS, and that this association cannot be explained by inhibitory control alone.

Supplementary table S1. Correlations among the examined variables, including results on the stop-signal task

	1	2	3	4	5	6	7	8	9
1. EL	-	-0.37**	-0.31**	-0.21*	0.84**	0.70**	0.05	0.05	0.01
2. LNS		-	0.53**	0.49**	-0.24*	-0.40**	0.33**	0.03	-0.15
3. SS			-	0.36**	-0.24*	-0.47**	0.36**	-0.05	-0.19
4. DS				-	-0.09	-0.19	0.14	-0.21*	-0.16
5. ODD					-	0.72**	0.15	0.08	0.01
6. ADHD						-	0.09	0.10	0.15
7. Age							-	0.18	-0.10
8. Gender								-	0.07
9. SST									-

*Note. EL = Score on the Emotional Control Scale of the BRIEF, LNS = Score on the Letter-Number Sequencing task, SS = Score on the Spatial Span backward condition, DS = Score on the Digit Span backward condition. ODD = Score on the oppositional defiant problems scale of the CBCL, ADHD = Score on the Attention Deficit/Hyperactivity Problems scale of the CBCL. SST= Stop-signal reaction time in the last half of the stop-signal task **. Correlation is significant at the 0.01 level (1-tailed).*. Correlation is significant at the 0.05 level (1-tailed).*
